# Actual biological diagnosis of acute myeloblastic leukemia in children

**Published:** 2014-06-25

**Authors:** V Buga Corbu, A Glűck, C Arion

**Affiliations:** * “Carol Davila" University of Medicine and Pharmacy, Bucharest; ** “I. C. Fundeni" Pediatrics Clinic, “Carol Davila" University of Medicine and Pharmacy, Bucharest

**Keywords:** acute myeloblastic leukemia in children, cytochemistry, morphology, immunophenotyping, cytogenetics

## Abstract

Abstract

Acute myeloblastic leukemia accounts for approximately 20% of acute leukemias in children. The days the microscope represented the main tool in the diagnosis and classification of Acute Myeloblastic Leukemia seem to be very far. This review summarizes the current diagnosis of this malignancy, where the morphological, cytochemical, immunophenotyping, cytogenetic and molecular characterization represents the basement of a risk group related therapy.

## Introduction

Acute myeloblastic leukemia (AML) which is also known as acute non-lymphoblastic leukemia, acute myeloid leukemia or acute granulocytic leukemia contains a heterogeneous group of hematologic neoplasia with different biological and clinical connotations, characterized by a blocking event in maturation and the proliferation of precursor myeloid cells.

 If AML is the most common form of leukemia in the adult, in children it is a rare disease, representing 15-20% of the acute leukemias in pediatric age, with a peak of incidence in the neonatal period and a relatively constant incidence during childhood, increasing during the teenage period [**1**].

Notions of pathogenesis 

 Acute myeloblastic leukemia is the result of a neoplastic transformation as a result of the disturbance of the proliferation and differentiation mechanisms of the stem cell which is oriented from the myeloid point of view.

**Fig. 1:**
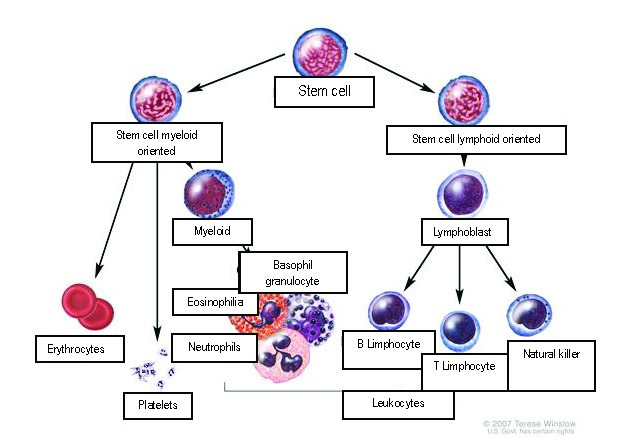
The stages of normal hematopoiesis

 Leukemogenesis is supposed to be a gradual process, which needs a susceptibility of the hematopoietic precursors to inductive agents. AML is a heterogeneous affection from the molecular point of view and the malign transformation can take place at different levels. 

 Many of the leukemic cells lose their apoptosis property, which has as a result a prolonged life cycle that implies a clonal proliferation without restrictions. Because the malignant transformed cells are not subject to regulatory mechanisms of the programmed cellular death anymore, they have priority in the competitive mechanisms with normal hematopoietic cells. The result is the accumulation of abnormal cells characterized by qualitative defects. The splenomegaly due to the leukemic infiltration also contributes to the pancytopenia, by seizing and destroying the erythrocytes and the platelets. The progressive substitution of the normal medullary population with leukemic cells leads to a spill of the blast cells in the peripheral circulation, with a possible infiltration of other organs. 

 The angiogenesis represents the process of forming new vessels from the preexistent vessels. The angiogenesis and the angiogenic factors have also proved to be important in the hematological malignancies not only in solid tumors. AML is associated with a growth of the vascularization in the bone marrow but also with a growth of the level of angiogenic factors such as VEGF, the fibroblastic growth factor, the derived growth factor of the platelets, the epidermal growth factor, TNF-alpha and beta, the hepatocyte growth factor (HGF), the angiogenic, angiopoietin-1 in the case of hematologic malignancies, most of these angiogenic factors seem to be secreted by hematopoietic neoplastic cells, assuring the growth and proliferation of the leukemic cells in an autocrine manner. VEGF and bFGF – the most powerful inductors of angiogenesis – are synthesized in leukemic cells but also in fibroblasts, immune cells, osteoclasts, platelets and megakaryocytes. Keith et al. showed that the gene expression of these factors is regulated in the bone marrow [**2,3**].

 From a pathogenic point of view AML is divided in primary forms (AML de novo) and secondary forms which developed consecutively to a myelodysplasia syndrome or related to the exposure to leukaemogenic agents (therapy related AML). 

 Although in the last decades, amazing progresses have been made regarding the discovery of the elements of pathogenesis in acute myeloblastic leukemia in children, there are still some questions that did not find an answer, such as: Which are the oncogenes that are important in AML? Do all the malignant cells have the same properties? Which is the role of the medullary microenvironment in AML? 

 Diagnosis work-up 

 • Anamnesis

 • Clinical examination + anthropometric indices 

 • Hemoleucogram + leukocyte formula, peripheral blood smear 

 • The examination of the bone marrow (cytology, cytochemistry) immunophenotyping, cytogenesis, molecular analysis) 

 • Lumbar puncture, if the patient is symptomatic 

 • Biochemical profile 

 • aPTT, PT, fibrinogen ( ± fibrin monomer, PDF)

 • Rx thorax 

 • Cardiac evaluation (ECG, Ultrasound) before the anthracycline treatment

 • Infectious status (VZV,CMV,HSV,HBV,HCV)

 • Sanguine group, Rh 

 • HLA typification, in patients who are possible candidates to hematopoietic stem cells transplant (HSCT)

 Diagnosis classification systems 

 Acute leukemias differentiate according to the type of proliferative cellular type, clinical manifestations, evolution and therapeutic answer; as a result, the possibility of diagnosing various types of diseases is crucial. 

 The first stage, which is mandatory, and which remains the cornerstone in establishing a certain diagnosis in acute myeloblastic leukemia but also in the exclusion of non-hematological proliferations and the lymphoproliferative diseases is represented by the cytomorphological examination. 

 The morphological analysis presupposes the use of blood smears obtained by marrow aspiration puncture and peripheral blood smears, colored in May-Grunwald-Giemsa. In most of the cases, the marrow preparation is hypercellular, with 80-100% atypical myeloblasts, but for the positive diagnosis a percent of 30% atypical myeloblasts is enough (according to FAB classification) or 20% atypical myeloblasts (according to WHO). 

 In cases of leukopenia, as well as in cases of uncertainty, the repetition of the aspiration puncture or the realizing of the bone biopsy puncture is indicated. The cytological framing using the French-American-British (FAB) system, considered the most complete morphological classification system, becomes more exact if it is completed by the immunohistochemistry criteria. Although this classification system represented the most important step in AML classification in children, the correlation with the prognostic indicators of survival and the answer to the treatment is limited. 

 FAB Classification System admits 8 subtypes of acute myeloblastic leukemia: 

 M0 – Undifferentiated AML – The blast population is made up of great myeloblasts, agranular which can sometimes be confused with the LAL-L2 ones. The diagnosis cannot be certainly established in optic microscopy but only based on demonstrating the presence of cytoplasmic granulations in electronic microscopy, of expressing some antigens belonging to the myeloid line by the blast cells, as well as expressing the absence of antigens specific to the lymphoid line. 

 M1 – AML without maturation – Blast population - 80-90% of the non-erythroid cells – is made up of large myeloblasts with rare azurophilic granulations. Auer corpuses are present in 50% of the cases. The diagnosis is positive if more than 3-5% of the cells react positively to MPO or Sudan black. The morphological study alone cannot differentiate this subtype of AML from LAL. 

 M2 – AML with maturation – 30-80% of the non-erythroid cells are very well differentiated myeloblasts characterized by a rich cytoplasm and a various number of azurophilic granulations. Auer corpuses are present in 65% of the cases. In 10% of the cells, the maturation exceeds the blast stage. The reaction for MPO is intensely positive, just like the one for Sudan black. 

 M3 – Promyelocytic acute leukemia – The myeloid population is dominated by the presence of pathologic promyelocytes, large cells with round nucleus, abundant cytoplasm, numerous great azurophilic granulations, with Auer corpuses placed “in bundles", sometimes masking the nucleus. The myeloperoxidase reaction is very intense. 

 M3V – Promyelocytic acute leukemia, microgranular version 

 M4 – Myelomonocytic acute leukemia – Precursors of the granulocytic and monocytic lines are present in the marrow in varying proportions, each line representing more than 20%, but not more than 80% of the nucleated cells. The myeloblasts contain Auer corpuses. The monoblasts are bigger than the myeloblasts, have a round nucleus, obvious nucleoli, abundant cytoplasm which contains azurophilic granulations and chromatin which is delicately dispersed. There is a high number of monocytes and promonocytes in the peripheral blood. The reaction for MPO and esterases completes the certitude diagnosis.

 M4eoz – Myelomonocytic acute leukemia with eozinophils – it has the same criteria as M4, but additionally, abnormal eozinophils with eozinophils and dysplastic basophilic granules are present. 

 M5 – Monoblastic acute leukemia – 80% of the non-erythroid cells are represented by monoblasts, promonocytes and monocytes. The reaction for MPO is negative, the reaction for nonspecific esterase is intensely positive. 

 M6 – Acute erythroleukemia (Di Guglielmo Leukemia) – The erythrocyte precursors represent over 50% of the medullary nucleate cells, with megaloblastic modifications and nuclear anomalies just like the ones in myelodysplasia. Over 20% of the nucleate cells are myeloblasts. The Auer corpuses can be met in 2/3 of the cases. From the cytochemical point of view, the erythroblasts are positive for the PAS reaction and esterase. 

 M7 – Megakaryoblastic myeloid leukemia (megakaryocytic) – numerous megakaryoblasts in different differentiation stages 

 - undifferentiated smaller forms, nucleus with dense chromatin, hardly visible nucleoli, reduced cytoplasm.

 - differentiated bigger forms, with delicate granular chromatin, abundant cytoplasm with azurophilic granulations. 

 The medullary product contains numerous reticulin fibers, which often makes the marrow puncture difficult to obtain. The diagnosis is specified by the cytochemical, immunophenotypical (the presence of specific markers) and ultrastructural (the presence of platelet chromatin peroxidase) examinations. 

 This subtype is frequently met in children with trisomy 21. 

 Histochemical analysis 

 The colors which are usually used – myeloperoxidase (MPO), periodical acid-Schiff (PAS), Sudan black B and esterases – find their utility in differentiating various subtypes of AML but also in differentiating AML from LAL in cases in which flow-cytometry studies offer doubtful results. 

**Table 1:**
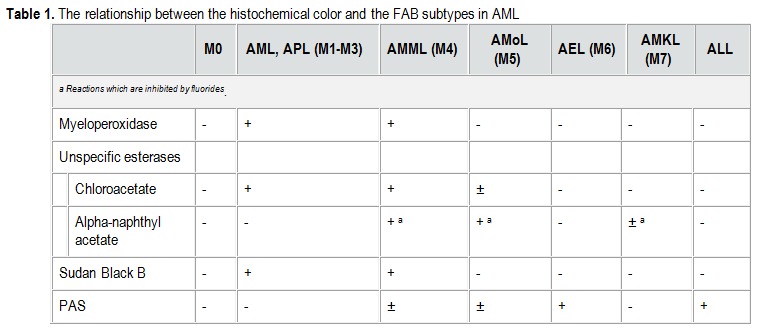
The relationship between the histochemical color and the FAB subtypes in AML

 The immunophenotypical analysis allows the differentiation of AML from LAL through flow-cytometry, according to the antigenic markers expressed at the surface or in the cytoplasm of the blasts and identifies different subtypes correspondent to the stages of cellular differentiation, markers which are reunited under the name of CD (cluster of differentiation-antigen).

 There are no antigens with absolute specificity or even more, there are some antigens that are characteristic to LAL, which can be also expressed in AML. The immunophenotypical characterization presupposes the use of some very large panels, which will include myeloid lineage markers (CD13, CD33, CD14, CD15, CDw41, CD11b, CD36 and glycophorin A), markers associated to line B (CD10, CD19, CD20, CD22 and CD24) and markers associated to line T (CD2, CD3, CD5 and CD7). HLA-DR (Human Leukocyte Antigen) testing proves its utility in identifying the LAM-M3 cases, whose positivity is rarely met in this subtype. Another practical application is the association of LAM-M7 with CD41, CD42 and CD61.

**Table 2:**
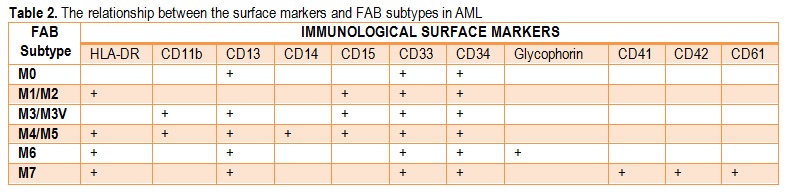
The relationship between the surface markers and FAB subtypes in AML

 The chromosomal and molecular biology analysis 

 Starting from the fact that the chromosomal and molecular biology particularities offer the most important prognostic information before the starting of the treatment, although the investigations are expensive, the purpose is that these modern methods are part of the basic diagnosis. 

 Molecular characterization: The biological material (minimum 5x10^6 cells) is processed in order to obtain the RNA. A molecular screening is done for AML1-ETO and CBFβ-MYH11 from the results of t(8;21)(q22;q22) and inv(16)(p13;q22)/t(16;16)(p13;q22), anomalies which have an important prognosis value. The criteria used in describing the cytogenetic clone and the karyotype follow the recommendations of the International Nomenclature System of Human Cytogenetics, the characterization of a normal chromosome needing the analysis of minimum 20 cells.

 What seems to be of great interest is the presence of t (15;17) responsible for the fusion of PML-RARα genes which are characteristic to the AML-M3 morphological form. In AML in children, mutations of the tyrosine kinase receptor are described; FLT3-ITD – identified in 16,5% of the cases – implies a negative prognosis of the disease. 

 WHO Classification (World Health Organization), which was first put to practice in 2002 and revised in 2008 has been the most complex until presently and significantly differs from the FAB system through the following: 

 • the reduction of the percent of marrow blasts which differentiate the myelodysplastic syndrome from AML, from 30% to 20%. 

 • the inclusion of cytogenetic criteria or the equivalent molecular anomalies in the classification. 

 • the inclusion of the cases preceded by myelodysplastic/chronic myeloproliferative syndromes in the classification. 

 • the inclusion of multilineage dysplasia with or without precedent marrow affecting in the classification. 

 • the inclusion of cases secondary to precedent cytotoxic therapy in the classification. 

 • the introduction of new morphologic subtypes. 

**Table 3:** WHO classification of acute myeloblastic leukemias (World Health Organization)

AML with recurrent genetic modifications	• AML with t(8;21)(q22;q22)/AML1(CBFA)-ETO • AML with marrow abnormal eosinophilia -inv(16)(p13;q22) -t(16;16)(p13;q22)/CBFB-MYH11 • APL M3(FAB) with t(15;17)(q22;q12)/PML- -RARA and variants
AML with multilineage dysplasia	• De novo / Secondary to myelodysplastic syndromes
Secondary AML	• Alkylating agents • Topoisomerase 2 inhibitors
Doubtful line AML	• Undifferentiated AML • Bilinear AML • Biphenotypic AML
Unclassifiable AML	• AML M0/M7(FAB) • Basophilic AML • Acute promyelocyte with myelofibrosis • Granulocytic sarcoma

 Defining the stages of the disease 

 ∎ Start: The AML diagnosis is defined according to WHO criteria – the presence of myeloid blasts in the marrow aspirate in a percent of at least 20% of the nucleate cells. 

 AML de novo: is the term that determines the form of the disease met in children who did not receive any treatment, except for the treatments that mark the symptoms of the disease. 

 AML secondary to a treatment (t-AML) or to any other affection. 

 ∎ Extramedullary disease: 

 It is well known that acute myeloblastic leukemia can invade the non-hematopoietic tissue (meninges, cerebral structures, abdominal organs, testicles, ovaries, skin, and ocular globes). Among the most important ones, the following should be mentioned: 

 1. SNC Affection – presupposes the presence of over 5 leucocytes/mmc of cephalorachidian liquid (CRL). If below 5 blast cells/mmc are present, the exploration must be repeated. The clinical neurological manifestations (epileptic seizures, affection of the cranial nerves, signs of cranial hypertension) which can be the expression of central nervous system involvement cannot be neglected. 

 2. Granulocytic sarcoma – presupposes the proliferation of myeloid cells at the level of the soft tissues without the involvement of the bone marrow, which can be identified through morphological methods. These forms need different diagnosis procedures and another therapeutic attitude. 

 ∎ Complete remission (CR) is defined by: 

 ∘ the presence of <5% myeloblasts in BM, in optical microscopy, in the conditions of a marrow cellularity of over 20% and at least 1500 circullant neutrophils with a duration of at least 4 weeks. The minimum residual disease (MRD/BRM) is defined as a cell mass of undetectable leukemic cells through usual morphological techniques. The immunological and molecular genetics techniques promise the evaluation of a more accurate minimum residual disease. What is studied is whether the action of defining CR through BRM is superior to defining it through optical microscopy. 

 ∘ lack of any symptom / sign connected to the disease 

 ∘ absence of the extramedullary disease and the SNC infiltration detected in the clinical examination and the CRL investigation. 

 ∎Partial remission: The presence of 5-20% myeloblasts in the blood marrow obtained by medullary aspiration at the end of an induction stage. 

 ∎Recurrent AML: The reappearance of the disease after obtaining a complete remission. 

 ∎Refractive AML: Failing to obtain a complete remission at the end of the induction stage, after applying the chemotherapeutical treatment according to current protocols (20% blast cells). 

 ∎CRL Remission: The patients with SNC involvement in the beginning stage are considered in CRL remission if 2 consecutive lumbar punctures indicate 5 leukocytes/mmc and do not show any blast cells as a result of centrifugation. 

 In conclusion, the remarkable progresses that have been obtained in the last couple of years in AML in children by adopting an individualized therapy, and which are adapted to risk groups, have as a base a set of diagnostic procedures. Although the classification of the patients on risk groups is influenced by many clinical and laboratory factors, the genetic modifications that represent the basis of pathogenesis are prominent in most of the classifications and are indispensible together with the immunophenotypical characterization in the current AML diagnosis in children. 
